# Cluster Modeling of Environmental and Occupational Health and Safety Management Systems for Integration Support

**DOI:** 10.3390/ijerph19116588

**Published:** 2022-05-28

**Authors:** Alena Pauliková, Jana Chovancová, Jarmila Blahová

**Affiliations:** 1 Institute of Industrial Engineering and Management, Faculty of Materials Science and Technology in Trnava, Slovak University of Technology in Bratislava, Jána Bottu 25, 917 24 Trnava, Slovakia; jarmila.blahova@stuba.sk; 2 Department of Management, Faculty of Management and Business, University of Prešov in Prešov, Konštantínova 16, 080 01 Prešov, Slovakia; jana.chovancova@unipo.sk

**Keywords:** environment, occupational health and safety, management systems, cluster, visualization, requirements, documented information, EMS, OHSMS, IMS, OH&S

## Abstract

Many organizations around the world recognize the complementarity of public and environmental health and focus their attention on the effective management of both health and environmental risks. For this purpose, they often use the international standards ISO 14001 and ISO 45001. However, when a company intends to implement multiple standards simultaneously, the challenge of overlapping increases. Therefore, the objective of article is to analyze the requirements and documented information of two management system standards: environmental management systems (EMS), according to ISO 14001, and occupational health and safety management systems (OH&S), according to ISO 45001. A combination of content analysis and clustering methods was used to conduct the research. Visualization of the interrelationships between the requirements of the standards was done using TouchGraph Navigator. The outputs of the analysis can serve managers in the integrated implementation of these management systems as well as auditors during the review and check process when formulating recommendations for the improvement of management systems. Integrated implementation comes with multiple benefits, including reduced bureaucracy and management costs, a simplified certification process, improved internal management, and facilitation of continuous improvement.

## 1. Introduction

Environmental risks and occupational accidents have essentially the same denominator: a certain degree of irregularity and randomness. However, their causes and consequences can be reduced by effective management systems that organize, simplify, and bring order and a certain structure to the management of the organization. This systems approach secures a better orientation within and compliance with the regulatory requirements, simplifying administration and saving financial sources [1]. To this end, the International Organization for Standardization (ISO) has developed a series of system standards dealing with environmental and occupational health and safety issues.

The ISO 14000 series of standards are devoted to environmental protection, with ISO 14001 being the core system standard. Occupational health and safety management is addressed in ISO 45001. The interconnectedness of these areas raises the question of the need for their joint management. Integrated management systems, also referred to as “multipart management systems” [2], are used to bring synergies and increase the effectiveness of environmental and occupational health and safety management. An integrated management system (IMS), as perceived in theory and practice, is based on a generic structure of international standards using a systems approach [3]. The generic design of management system standards is the central aspect of their integration; i.e., their ability to merge into a single, integrated management system based on processes. The latest revision of the main system standards ISO 9001 and ISO 14001, which took place in 2015, and ISO 45001 in 2018, has brought a significant shift towards facilitating the integration of management systems, as it applies common requirements and a so-called high-level structure (HLS, Annex SL) with which management systems must comply [4].

However, several mandatory requirements and documented information set out in these standards overlap, which increases the likelihood of duplicity, bureaucracy, and inefficiency. Therefore, the present paper aims to analyze both standards’ common and specific requirements to facilitate their integrated implementation. For this purpose, a combination of analytical and visualization tools will be used, including content analysis, cluster modeling, and Touch graph navigator. The outputs aim to contribute to more efficient implementation and auditing of these management systems.

Despite numerous studies focusing on the integrated implementation of health and safety and environmental management systems, and their anchoring in global strategic documents, there is not yet a coherent and managerially effective overview of the common requirements as well as the specific requirements of the two standards in the literature. For this reason, the present article aims to analyze the common elements and differences/specific requirements of the two ISO standards and propose a couple cluster to facilitate their integration at the organizational level. The novelty of this approach lies in (1) the identification of common and specific requirements of environmental and health and safety management systems to eliminate duplicities during the integration process; (2) the visualization of these requirements for clarity and transparency for managers and auditors; and (3) application of new methods of analysis and visualization, which can also be used in the integration of other management systems.

The paper is organized as follows: first, both management systems (environmental and occupational health and safety) and their reference documents are introduced; the second section presents a literature review; the third section describes the methods used (content analysis and clustering); the fourth section presents the obtained results; the fifth section contains the discussion of the results and outlines the future pathways and limitations of the research; and the final section concludes the article.

## 2. Literature Review

The benefits associated with the integration of management systems are reported in many empirical studies. An integrated management system is an overarching management system, which considers the various elements, functions, and perspectives of a company’s organization holistically. This includes business functions, such as human resources, finance management, marketing management, production management, etc., and cross-sectional functions, such as quality management, environmental management, work safety management, and others [5]. According to [6], proper integration of management systems is accompanied by decreased bureaucracy, management costs, a simplified certification process, better internal management, and facilitation of continuous improvement. Cost savings, better use of resources, improved internal communication, stronger customer orientation, and employee motivation are the benefits of IMS reported by airline companies [7]. The multiple benefits of implementing integrated management systems are summarized in a recent study [8], which provides evidence of the importance of integrated management systems in facilitating monitoring, assessment, and evaluation of the effectiveness of management systems via integrated audits, increasing the credibility of the organization and generating new business opportunities [9]. Although research on the impact of IMS on organizational financial performance has produced mixed and ambiguous results (see, e.g., [10,11,12]), the financial link to environmental and occupational health issues is strong [13]. As [14] states, work-related accidents and illnesses cost the EU at least EUR 476 billion every year, and global costs of EUR 2680 billion represent 3.9% of global GDP. Nor is the environmental consequence negligible: It is estimated that, in 2019, a total of 368,006 premature deaths occurred in Europe due to air pollution [15]. Moreover, as [16] noted in his report, ignoring climate change can destroy economic growth.

An integrated approach to environmental and health risk management is evident in several studies. Tepaskoualos and Chountalas demonstrate the successful outcome of integration in the case of a construction company [17]. The benefits of simultaneous implementation of occupational health and safety and environmental management systems, with the support of a management information system, were highlighted in the case of a nuclear power plant in Eastern China [18]. The specific situation of small and medium-sized enterprises in implementing EMS and HSMS in Thailand is addressed in a study by Jaroenroy and Chompunth. They propose a conceptual framework for an alternative integration of these two systems, taking into account the characteristics of small and medium-sized steel manufacturers [19].

Kruse et al. focused on integrating health and safety and environmental management systems and presented an integrated lean management system framework and the strategies available and used by a sample of high technology performance organizations to simultaneously protect workers, the environment, and support lean enterprise outcomes [2].

The integration of environmental and occupational health and safety management systems has a broader global context, as indicated by the overlap with the Sustainable Development Goals defined by the United Nations in 2015 (Table 1). ISO 14001 is related to 12 SDGs (70.6%) and ISO 45001 is related to 7 SDGs (41.2%).

If we look more closely at the impact of the two standards acting integrally, we can see that they encompass almost all SDGs, i.e., 16 out of 17 SDGs (94.1%), and both standards and SDGs are inextricably intertwined [21]. Therefore, it can be assumed, and practice confirms it, that the integration of environmental and health and safety management systems provides the opportunity for sustainable development of the organization and society.

Many organizations around the world follow the integration rules. Companies’ management systems bring advantages in terms of:

- Enhance the reputation of the organization to their interested parties;
- Contribute to an integrated approach to risk management in business;
- Increase the capacity of the organization to attain objectives;
- Provide better alignment of strategic, tactical, and operational policies and objectives;
- Provide competitive advantages from synergies of different management policies;
- Eliminate hostilities, doubt and redundancy among the management standards;
- IMS is helpful to attain sustainability in business;
- Improvement of organizational and social culture [22];
- Improvement of productivity and organization efficiency;
- Multiple systems with the same goals;
- Reduction of management costs and impacts [23];
- Better definition of management responsibilities and authority;
- Improved external image of the organization [24];
- Strong tools for environmentally oriented business [25];
- The ability to ensure that the business is sustainable and takes full advantage of business opportunities [26].

Overall, the management systems are implemented as integrated management systems in order to achieve a unified organizational structure for sustainability [27]. Their main goal is to create a sustainable and flexible structure that will respond to change and at the same time take into account many types of requirements. To avoid the thematic voids or waste of operational resources and to reach a more holistic and interactive way of sustainability management, an integrated management system (IMS) is necessary [28,29,30].

## 3. Materials and Methods

### 3.1. Characteristics of Environmental and Health and Safety Management Systems

An environmental management system, EMS, helps organizations identify, manage, monitor, and control their environmental issues in a “holistic” manner. The ISO 14001 Environmental Management Systems—Requirements with Guidance for Use is globally the most widespread standard related to environmental management systems [18,19].

ISO 14001 is an internationally agreed standard that sets out the requirements for an EMS. This standard supports any organization to improve their environmental performance through more efficient use of resources and waste reduction, gaining a competitive advantage and the trust of interested parties. Enterprises applying ISO 14001 report success across of various areas, including reduced water and energy consumption, a more systematic approach to legal compliance, and an improved overall environmental performance [31].

Achieving accredited certification according to ISO 14001 certainly delivers commercial value to an organization, including reduced greenhouse gas emissions and streamlined waste management, and provides a better handle on business risk and competitive advantage. Thus, the implementation of EMS creates a win–win effect for the economic performance of the organization as well as a better quality environment [32].

An important concern of everyone in an organization, whether owner, manager, or worker, is that no one gets hurt on the job [32]. According to the International Labor Organization (ILO), there are currently more than 2.78 million deaths a year because of occupational accidents or work-related diseases, in addition to 374 million non-fatal injuries and illnesses.

Aside from the enormous impact on families and communities, the cost to businesses and economies is significant. ISO 45001, Occupational health and safety management systems—Requirements with guidance for use [33], is the world’s first international standard for occupational health and safety (OH&S). It provides a framework to increase safety, reduce workplace risks, and enhance health and well-being at work, enabling an organization to proactively improve its OH&S performance [34].

The main managerial reasons and information about these two standards for effective implementation are summarized in Table 2.

A Type A standard is a type of standard that contains requirements against which an organization can claim conformance. To claim the conformance, the organization must provide evidence to support that the organization meets the requirements specified in the standard. Evidence is usually obtained through an audit process. This can only be done based on a document that contains these requirements.

Harmonized structure (HS) of the standard means that the standard has the same structure and contains many of the same terms and definitions. This is particularly useful for those organizations that choose to operate a single (sometimes called “integrated”) management system that can meet the requirements of two or more management system standards simultaneously [35].

### 3.2. Data Analysis for Double Cluster Tabulation

A combination of content analysis and cluster analysis was used to analyze the common and specific requirements and documented information of the two standards (ISO 14001 and ISO 45001). Both methods are widely used in qualitative research. Content analysis is a research technique for making valid, replicable, and objective inferences based on explicit rules from the text (document) [36]. Various scholars have described the methodological aspects of content analysis, including [37,38,39]. This method has been used extensively in research in many fields, including health and medicine (e.g., [40]), public relations (e.g., [41]), legislative studies (e.g., [42]), marketing (e.g., [43,44]), business ethics (e.g., [45]), and others.

The logic and composition of the research procedure consisted of the following steps:

1. Identifying and data/material collection—for content analysis, two sources of data, i.e., input documents, were selected: the ISO 14001 and ISO 45001 standards;
2. Creating a codebook—a codebook containing the list of codes for data analysis was created. It contained two codes and code definitions: (1) Shall—the verbal form used in ISO standards indicating a requirement. The requirement is a need or expectation that is stated, generally implied, or mandatory. (2) Documented information—indicating the information required to be controlled and maintained by an organization including the medium on which it is contained (any format and media). As the standard is stringent in its use of terminology, additional codes have not been selected to denote requirements;
3. Coding the content—in Clauses 4–10 in both standards, the sections corresponding to the code list were highlighted;
4. Recording incidences and frequency—a frequency count on how many incidents occurred for each code item in each clause was performed. This was followed by a revision of the text and the numerical identification of each requirement and documented information;
5. Comparison of the coded data—the correspondence/similarity of the requirements in the highlighted text of each clause for ISO 14001 and ISO 45001 was compared (comparisons are made for clauses, subclauses, sub(2)clauses, etc.). The occurrence of a match was recorded in a pre-established form;
6. Checking validity and reliability—a revision must be made with comprehension and a sense of detail and accuracy of interpretation. Despite HLS or HS (harmonized structure) standards, the overlap is possible. This means, for example, that for Subclause 5.4 for ISO 45001, the exact requirements occur in Subclause 6.1.1 for ISO 14001. Therefore, the comparison of requirements has been adapted to the content specificities of these standards. It should also be noted that the number of sub-clauses varies for each management system according to the ISO standards. These differences also occur for other system standards, which should be considered when carrying out content analysis;
7. Final analysis and summary—the occurrences of all common requirements and the occurrences of all common documented information for all clauses for ISO 14001 and ISO 45001 were counted and prepared for cluster modeling.

After completing the 7th step, it is possible to perform the double cluster tabulation, i.e., to insert data into a final file. Supported input formats include Excel, Csv, Tsv, Mulli Csv, MySql, Na DB API, Vna, Ddf, and Pajek. In our case, we used an Excel (.xls) file to create a double cluster of the management systems standards. The order of the data in the rows and columns may vary. It depends on the user which type of layout will be used.

### 3.3. Application of Data File for Double Cluster Visualization

The visualization of clusters is progressively applied to illustrate variants of models ranging from societies and relationships to hierarchies or spatial networks. The comprehensive technique of visualization includes the following phases:

- Data selection, transformation, tabulation, and data import;
- Origination of points and interconnection and their formation to highlight the characteristics of the cluster;
- Assigning unique attributes to make better visual effects (e.g., color, size of points, interconnections);
- Study of visualized networks—interactive work with the model.
- For the design of this network, we chose the software TouchGraph Navigator to build the management systems standards visualization or double cluster. This program makes it possible in a simple way to build a special interactive network of accessible data, which can be loaded in several formats.

This visualization software can analyze and present relations by arranging the model appearance and filter settings, export figures and data, and save the resulting model so that the outputs can be shared and start back exactly where you left off [46].

## 4. Results

Cluster modeling is a stepwise process. A systematic approach is essential to ensure that all necessary data inputs are included in the model. It is recommended to perform an inter re-verification of the correctness and functionality of the model after each step. The modeling skills of integrated management systems increase with each subsequent project [47,48].

### 4.1. From 0th to 2nd Degrees of Separation—From the Centre to the Main Clauses

The initial stage in building a cluster is to create the first center or unit named “CLUSTER MSSs” (black color rounded rectangle with grey color aureole). This center has its *0th degree of Separation*. The follow-up stage covers the pair of units for two standards: “ISO 14001:2015 EMS” (green color rounded rectangle with an earth tag in the upper left corner) and “ISO 45001:2018 OHSMS” node (dark green color rounded rectangle with two figures tag in the upper left corner) with *1st degree of Separation*. The pair of management systems standards present the fundamentals of the cluster MSSs.

**Figure 1:**
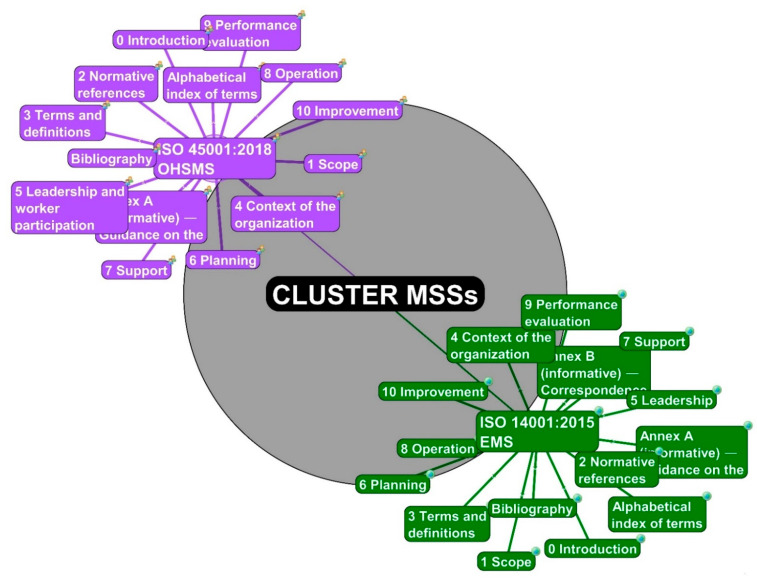
The center units with pairs of standards and their main clauses (source: authors).

Both standards have their clauses. According to the harmonized structure for management systems standards, there are ten main clauses. The standard ISO 14001 contains *Annex A* and *B* as well as an *Alphabetical index of terms*. The standards ISO 45001 have only *Annex A* and *Alphabetical index of terms*. The chapters *Annexes* and *Alphabetical index of terms* have equal status in clusters as clauses and are activated with *2nd degree of Separation*, Figure 1.

The color of the individual standards, clauses, and sub-clauses is fixed for easy differentiation and affiliation determination. The focal point and the standards are logically linked. This procedure of connecting individual points is used throughout the cluster.

Both standards, including clauses and subclauses up to the fifth structuring, (0; … A.8.1.4.3, … B, Alphabetical index of terms), relate to lines that vary in their thickness, pattern, and color.

### 4.2. From 3rd to 4th Degrees of Separation—From PDCA Cycle to the Requirements

**Figure 2:**
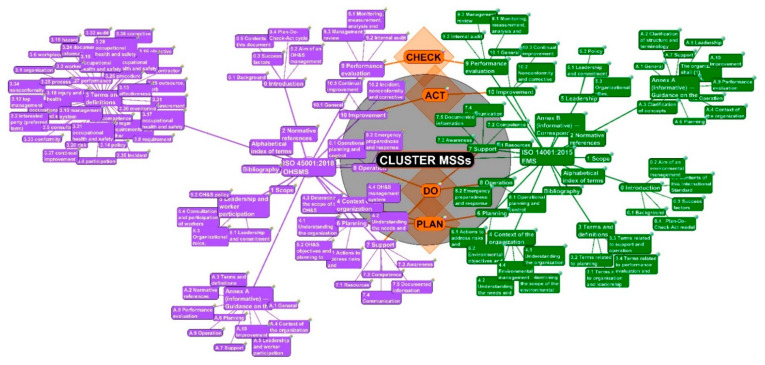
An activated PDCA cycle with four points, PLAN, DO, CHECK, and ACT, for both MSSs with their connections (source: authors).

The PDCA cycles are visualized with subclauses with the 3rd degree of Separation activation in Figure 2.

The diamond points, “PLAN”, “DO”, “CHECK” and “ACT”, are presented in orange color and with a rhombus shape and rainbow circle tag. These entities are connected according to their affiliation in the framework of the harmonized structure of a couple of MSSs (ISO 14001:2015 EMS, ISO 45001:2018 OHSMS). The point “PLAN” is linked with “Subclauses 4.1, 4.2, and Clause 6” for both standards and Subclause 5.4 for OHSMS. The entity “DO” is connected to “Clauses 7 and 8” for both standards. The point “CHECK” is linked with “Clauses 9” and, finally, the point “ACT” relates to “Clauses 10” for both MSSs. These links can be verified in Table 3 as well. 

In the 3rd degree, the subclauses of both standards are activated. Subsequently, the sub-sub clauses (sub(2) clauses) are shown in the *4th degree of Separation*, followed by the sub-sub-subclauses (sub(3)clauses) in the *5th one* and the end by sub-sub-sub-subclauses (sub(4)clauses) in *6th degree of Separation*. The cluster creates its unique complex. 

Suppose an enterprise that manufactures products and provides services, or any organization, decides to implement management systems per the relevant standard. In that case, it must prepare a list of mandatory requirements that, when met, demonstrate the correctness of implementation.

The set of mandatory requirements consists of the requirements of management systems according to the relevant ISO standard, legal regulations, internal regulations, customers’ requirements, and others. The requirements in the standard are not highlighted and therefore it is necessary that the implementers to gradually search for and list them in the text. This is a very demanding process for understanding management systems, for the ability to read with understanding, and is a time-consuming process. All requirements are indicated using the verbal form “shall” in the management systems standard text.

The mandatory requirements in our cluster, initialized from *the 4th degree of Separation*, are shown by the red color (for ISO 14001) and the dark-red color (for ISO 45001) rounded rectangular with an anchor tag, with the halo size effect in the shape of a square.

**Figure 3:**
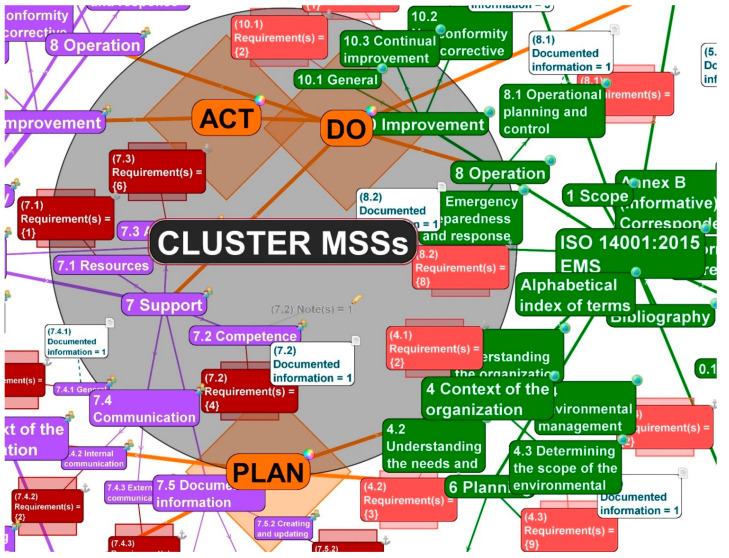
Requirements and documented information of ISO 14001 EMS and ISO 45001 OHSMS, both MSSs with their connections, in the cut-out (source: authors).

Furthermore, the connections leading from the requirements to a relevant point (clause) have an identical color to the requirement of ISO 14001 or ISO 45001. In (dark) red rectangular shapes there are several mandatory requirements written in composing brackets for each clause, subclause, etc. (see Figure 3).

### 4.3. From 5th to 6th Degrees of Separation—From Documented Information to Notes

Evidence-based and data-driven decision making is crucial for managers to drive effective action in an organization. Therefore, mandatory requirements need to be documented. ISO standards for management systems keep this in mind by providing, exhaustively, the existing documented information in the text. The documented information is equally important when viewed in our cluster. Documented information is displayed in a white, rounded rectangular, with the white page tag. In the white rectangular, there is several documented information written for each clause, subclause, etc. (see Figure 3).

**Figure 4:**
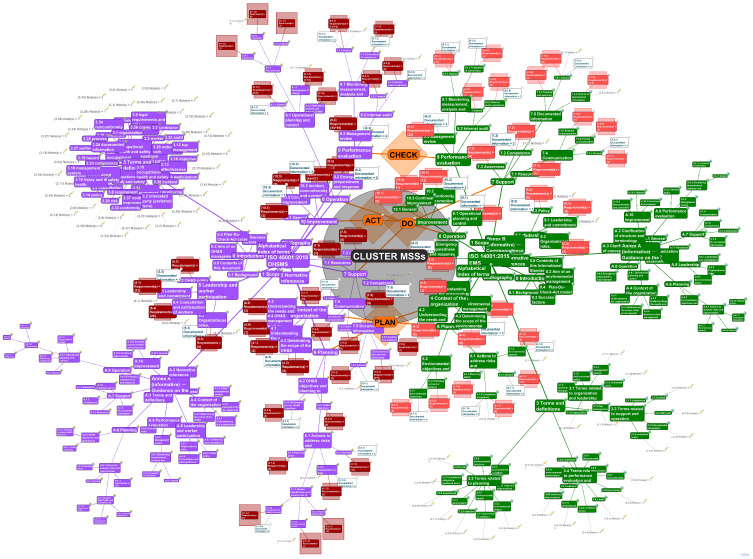
Complex visualized double cluster for EMS and OHSMS with the affiliated requirements, documented information, and notes (source: authors).

A comprehensive overview of the whole model is shown in Figure 4.

With the correct integration of management systems according to the ISO 14001 and 45001 standards, it is reasonable and essential to find out in advance which requirements and documented information are common and which are different, i.e., specific. This leads to the merging of both management systems as much as possible.

To explain the mandatory requirements, management systems according to ISO standards contain an additional tool, “Notes”. These comments can help implement MSSs. In our cluster, these explanations are included, visualized in a text line, indicating the number of notes for each clause, and have a pencil tag assigned to them.

Figure 4 illustrates the complex visualized cluster for EMS and OHSMS with their affiliated requirements, documented information, and notes. In the complex model, *5th and 6th degrees of Separation* were activated to display the 4th and 5th level of clauses (sub(4) and sub(5) clauses, e.g., up to A.8.1.4.1).

**Figure 5:**
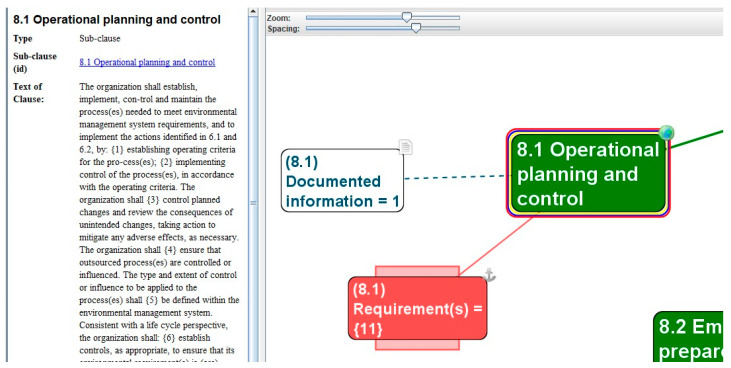
Overview of all the requirements {11} and Documented information/1 of Subclause 8.1—Operational planning and control for the ISO 14001 EMS, using a scan from the software TouchGraph Navigator (the cut-out) (source: authors).

By activating the individual clause (sub(x)clause), a text field is displayed in the TouchGraph Navigator software application with the full wording of the entire clause (sub(x)clause) and an indication of the mandatory requirements in composing braces. In this way, implementers, managers, or any users of the MSS can gain immediate access to information about the mandatory requirements, their numbers, and the text containing the requirement of the documented information and their number, and can read the explanatory note. Figure 5 illustrates an example of an overview of all the requirements {11} and Documented information/1 of Subclause 8.1—Operational planning and control for the ISO 14001 EMS.

### 4.4. Integrated Management Systems Standards—Couple Model of EMS and OHSMS

The integration of EMS and OHSMS can be performed as an organizational project that refers to the implementation of a pair of standards and the integration of the related mandatory requirements and documented information into one integrated management system [49].

Table 3 presents several clauses (subclauses, sub(2)clauses, sub(3)clauses, sub(4)clauses) with affiliation to the PDCA cycle. For each EMS/OHSMS (sub)clause, the number of mandatory requirements is given in composing brackets and the number of documented information is provided after the backslash. The last column of Table 3 lists the common requirements for both management systems according to both ISO standards. This forms the basis to facilitate the proper and correct integration of ISO 14001 EMS and ISO 45001 OHSMS.

In Table 3, there are several requirements, {R}, in composed brackets, and documented information, DI, for the management system standards ISO 14001: 2015 EMS and ISO 45001: 2018 OHSMS, as well as their common {R} and DI.

The total number of EMS requirements is 188 ÷ 194 along with 23 documented information. (The interval indicates the minimum and the maximum number of requirements; it depends on the context of the organization and its stakeholders.) The total number of requirements of OHSMS is 271 ÷ 284 and 27 documented information. The number of common requirements for integrating EMS + OHSMS is 142 ÷ 148, with 16 common documented information.

**Figure 6:**
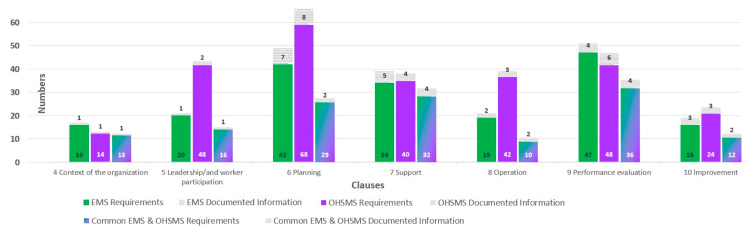
Number of requirements and documented information for EMS and OHSMS (source: authors).

An overview graph of the requirements and documented information for the clauses of the EMS and OHSMS standards and their integrated requirements and documented information is shown in Figure 6.

**Figure 7:**
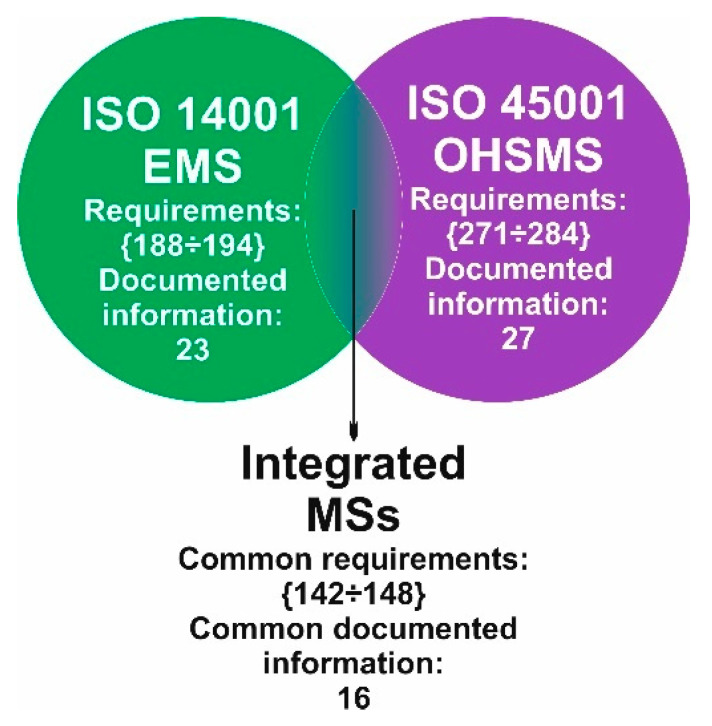
The intersection of EMS and OHSMS for visualized integration with the total number of common mandatory requirements and documented information (source: authors).

Figure 7 illustrates the intersection between EMS and OHSMS for visualized integration with a total number of common mandatory requirements and documented information.

## 5. Discussion

The standard ISO 45001:2018 significantly supports organizations serious about improving employees’ safety, reducing workplace risks, and creating more suitable, safer working conditions. The OH&S standard shares the identical base text of the document, terms, and definitions, and a high-level structure with the revised ISO management systems standard ISO 14001:2015. 

This framework is designed to facilitate the integration of the management topics into an organization’s established management systems. In addition, ISO 45001 was designed to follow ISO 14001 closely, as it is recognized that many enterprises combine their OH&S and environmental management functions internally. If interested parties are familiar with ISO 14001 then the integration with ISO 45001 will be much easier to perform [34].

In the future, it would be possible to develop and supplement the created cluster with other management systems according to ISO standards. When analyzing the content of the given systems, it is necessary to think in advance about their implementation, maintenance, and improvement. For further research activities, it seems helpful to add application standards with guidelines for using the EMS and OHSMS couple:

- ISO 14002-1:2019 Environmental management systems—Guidelines for using ISO 14001 to address environmental aspects and conditions within an environmental topic area—Part 1: General [50];
- ISO/DIS 14002-2 Environmental management systems—Guidelines for using ISO 14001 to address environmental aspects and conditions within an environmental topic area—Part 2: Water [51];
- ISO 14004:2016 Environmental management systems—General guidelines on implementation [52];
- ISO 14005:2019 Environmental management systems—Guidelines for a flexible approach to phased implementation [53];
- ISO 14006:2020 Environmental management systems—Guidelines for incorporating ecodesign [54];
- ISO 14009:2020 Environmental management systems—Guidelines for incorporating material circulation in design and development [55];
- ISO/DIS 45002 Occupational health and safety management systems—General guidelines for the implementation of ISO 45001:2018 [56];
- ISO 45003:2021 Occupational health and safety management—Psychological health and safety at work—Guidelines for managing psychosocial risks [57];

When analyzing the content of the standard, it is necessary to recognize that some management systems standards define the requirements, which are always stated in the standard with the modal verb “shall”. Other management standards provide a step-by-step guide for implementing the given management systems according to the ISO standard; i.e., they contain guidelines with the modal verb “should”. The third type includes both requirements and guidelines, so the use of the modal verb “shall” when searching for mandatory requirements and documented information applies to this type. The procedure for analyzing the content of a given standard must be adapted to the purpose of further use of the standard.

Therefore, the “decoding” of the content of the standard must also include other keywords corresponding to the recommendations, because the principal auxiliary verb, “should”, in standards with requirements is represented by the verb “shall”. This requires careful reading with understanding, often repetitive, of individual clauses and subclauses.

ISO 14001 and 45001 standards are widely applicable in various industries that deal with manufacturing or service delivery. They are also tied to the manufacturing companies in the automotive industry. For these companies, it is possible to cluster a pair of ISO 14001 and ISO 45001 standards with the following management systems:

- ISO 9001:2015 Quality Management Systems—Requirements [58];
- IATF 16949:2016 Automotive Quality Management System Standard, Quality management system requirements for automotive production and relevant service parts organizations [59];

In food companies, it is possible to expand our cluster with other management systems according to [60]:

- ISO 22000:2018 Food safety management systems—Requirements for any organization in the food chain [61];

In the energy industry:

- ISO 50001:2018; Energy management systems—Requirements with guidance for use [62];
- ISO 19443:2018 Quality management systems—Specific requirements for the application of ISO 9001:2015 by organizations in the supply chain of the nuclear energy sector supplying products and services important to nuclear safety (ITNS) In petroleum, petrochemical, and natural gas industries [63];
- ISO 29001:2020 Petroleum, petrochemical and natural gas industries—Sector-specific quality management systems—Requirements for product and service supply organizations [64];

For the water supply industry:

- ISO 46001:2019 Water efficiency management systems—Requirements with guidance for use [48,65];

For supply chain organizations:

- ISO 28001:2007 Security management systems for the supply chain—Best practices for implementing supply chain security, assessments and plans—Requirements and guidance [66];
- ISO 28002:2011 Security management systems for the supply chain—Development of resilience in the supply chain—Requirements with guidance for use [67];

In transport services:

- ISO 39001:2012 Road traffic safety (RTS) management systems—Requirements with guidance for use [68];
- ISO/TS 22163:2017 Railway applications—Quality management system—Business management system requirements for rail organizations: ISO 9001:2015 and particular requirements for application in the rail sector [69];

For laboratories:

- ISO 35001:2019 Biorisk management for laboratories and other related organizations [70];

Overall, in society, public and state administration:

- ISO 26000:2010 Guidance on social responsibility [71];

Regarding the risk:

- ISO 31000:2018 Risk management—Guidelines [72].

It is also interesting to combine this cluster with other models that work on different principles; e.g., the fuzzy model of risk assessment for environmental start-up projects in the air transport sector. These combined models increase, during a more significant implementation, the degree of validity of the decision making regarding the financing of the investors by the investing publicly at the stage of market expansion [73].

### Limitations and Future Research

The research limits occurred when modeling clusters of management systems forced us to perform a vertical and horizontal analysis of the standard text. Even with a high-level structure (HLS) or harmonized structure (HS), it is necessary to compare not only the assigned clause number but also the content of the text. To correctly integrate the management systems standards, it is essential to perform a comprehensive reading, focused comparison, and detailed search for binding common and specific requirements. For example, Table 3 shows the 11 requirements of Subclause 8.1.—Operation Planning and Control of ISO 14001. Of these 11 requirements, 6 can be found in Sub(2)clause 8.1.1.—General in ISO 45001, one level down. Hence the reason for creating an overview that is not only horizontal but also vertical. 

Based on an extensive literature review, we identified other possible limitations and challenges of the proposed methodology. Depending on the size of the organization and its business form, data management might be a challenge [74]. Regular standard updates can cause particular challenges, especially when combined into one IMS. Another challenge can be found in deciding on the proper integration mechanism. Part of the decision-making process is also the need to avoid bias against one particular system [75].

If the companies’ strategy is to implement more than one management system, there is a clear advantage of doing it supported by an integrated approach, avoiding the development of organizational “islands” related to each subsystem. This organizational “archipelago” structure is completely far from way from any global optimized solution, based on a holistic perspective [76].

The cluster with its dynamic and logical structure, mandatory requirements, and documented information is very helpful in the managerial decision-making process, for the friendlier study of individual clauses, and for conducting audits using ISO 19011 Guidelines for Auditing Management Systems if the audit criterion is a given EMS and OHSMS management system [77].

Future applications and research are derived from current challenges and should focus on how to integrate individual standards. It is necessary to find a way to incorporate the standards based on the system’s type, content, or size. Is it possible to integrate systems at once or gradually? Is it necessary to integrate entire systems or can you choose only the part of the standard that is specifically related to the issue? How integration will be approached in the future depends on the flexibility of the system and related requirements. From a business viewpoint, sustainability means “a business approach that creates long-term shareholder value by embracing opportunities and managing risks coming from economic, environmental and social developments.” [78].

## 6. Conclusions

The Delphi study [19] confirmed the need for merging of specific elements that overlap in the standalone management systems. For example, 100% of panel members suggested the elements of policy requirements, risk identification and assessment, competency training and awareness, operational hazard control, emergency preparedness and response, corrective and prevention action, incident investigation, and management review overlap should be merged. If it comes to other parts of the systems, e.g., evaluation of compliance, legal and other requirements, objectives, targets and improvement and internal and external audit, roles, and responsibilities, over 90% of panel members suggested that the elements overlap in the systems and suggest merging.

Implementing integrated management systems is of interest to experts in the academic field and practitioners in business and management regarding the previous paragraph. The subject of interest of the present article was the analysis of requirements and documented information of two frequently used management systems: EMS, according to ISO 14001, and OHSMS, according to ISO 45000. Integrating these two standards is based on the complementarity of public and environmental health. Just as in the real world, these two dimensions are intertwined and interdependent, so it is in a business where the joint implementation of environmental and occupational health and safety management systems creates an invaluable synergistic effect.

The analysis and subsequent comparison and synthesis provided an overview of those requirements and documented the common (intersectional) information that is found in environmental and occupational health and safety management systems. The summary of these common requirements and documented information will facilitate their integrated implementation in enterprises, associated with several benefits, including a reduction in administrative burden on employees, reduction in duplication in work and documentation, increased clarity and information in management decision-making, easier and systematic auditing process of the integrated management system, etc. In a broader perspective, the joint implementation of environmental and occupational health and safety management systems has the potential to contribute significantly to the achievement of sustainable development goals.

A combination of content analysis and clustering methods was used to conduct the research. Visualization of the interrelationships between the requirements of the standards was done using TouchGraph Navigator. The results demonstrate the validity of these methods and tools and create a prerequisite for their wide application in identifying the common elements of other management systems that an organization wants to use to improve the management of its processes.

### 6.1. Practical Benefits of the Study

Companies are often required to obtain ISO certification to enhance their competitiveness in the market and comply with stakeholder requests. Implementing one system is already a complicated task and depending on the size and the purpose of the company, it can take time and significant resources. The methodology proposed in this study considerably shortens the time needed to implement not only one but multiple systems simultaneously. The study serves as background material for the management team in any organization that manufactures products or provides services and plans to (1) implement, (2) verify viability, and (3) improve its integrated management systems following ISO 14001 and ISO 45001. It offers an enhanced ability to work with documents in the so-called autonomous regime; i.e., even if there is no integration between ISO 14001 and ISO 45001, it is possible to consider the requirements and prepare documented information for each standard separately.

Furthermore, using the visualization methodology, we tackle the complexity of the integration by providing a quick insight, a real-time schedule auditing of the integrated system, and rapid orientation in the links between clauses, requirements, and documented information.

IMS implementation is well known for the amount of paperwork associated with the process. The methodology advises on a precise numerical expression of the requirements and documented information for ISO 14001, ISO 45001, and IMS. Because of its flexibility, the process provides fast and efficient simplification adaptation of the entire integrated management system to the organization’s specific conditions, e.g., exclusion of requirements and documented information that is irrelevant to the given organization by hiding unrelated individual nodes in the cluster.

Finally, the illustrated method provides a possibility to develop a visualized cluster-integrated system from other points of view, e.g., processes, products, services, risks, opportunities, augmented reality, and others, according to the organization’s business portfolio.

### 6.2. Benefits of the Study for Research and Education

The proposed approach assures the ability of the organization to find integration “weaknesses” in the ISO 14001 and ISO 45001 standards. Visualization and clustering, as a methodology, offer additional support to integration and provide a new understanding of the interaction between clauses, requirements, and documented information. It can also be stated that this approach creates a platform for adding legislative requirements at the national/international levels for ISO 14001 and ISO 45001 by adding new nodes and interconnections between existing ones.

The visualization of the interconnection between the PDCA cycle and individual clauses is a powerful management tool. The cluster enables work with individual groupings and the possibility to separate the given area for further research; e.g., if the environment and the working environment converge.

Finally, clustering is a learning tool for bachelor’s, engineering, and doctoral students.

## Figures and Tables

**Table 1:** ISO 14001 and 45001 contribute to the following Sustainable Development Goals (Adapted with permission from [20] by the authors).

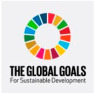	Sustainable Development Goals	ISO 14001	ISO 45001
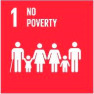	Goal 1: No Poverty End poverty in all its forms everywhere	YES	
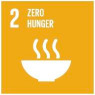	Goal 2: Zero Hunger End hunger, achieve food security and improved nutrition and promote sustainable agriculture	YES	
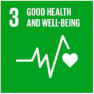	Goal 3: Good Health and Well-being Ensure healthy lives and promote well-being for all at all ages *	YES	YES
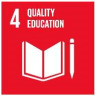	Goal 4: Quality Education Ensure inclusive and equitable quality education and promote lifelong learning opportunities for all	YES	
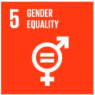	Goal 5: Gender Equality Achieve gender equality and empower all women and girls		YES
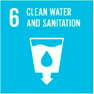	Goal 6: Clean Water and Sanitation Ensure availability and sustainable management of water and sanitation for all	YES	
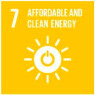	Goal 7: Affordable and Clean Energy Ensure access to affordable, reliable, sustainable and modern energy for all	YES	
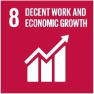	Goal 8: Decent Work and Economic Growth Promote sustained, inclusive and sustainable economic growth, full and productive employment and decent work for all *	YES	YES
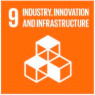	Goal 9: Industry, Innovation and Infrastructure Build resilient infrastructure, promote inclusive and sustainable industrialization and foster innovation *	YES	YES
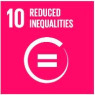	Goal 10: Reduced Inequalities Reduce inequality within and among countries		YES
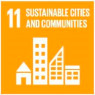	Goal 11: Sustainable Cities and Communities Make cities and human settlements inclusive, safe, resilient and sustainable		YES
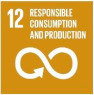	Goal 12: Responsible Consumption and Production Ensure sustainable consumption and production patterns	YES	
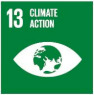	Goal 13: Climate Action Take urgent action to combat climate change and its impacts	YES	
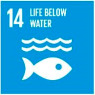	Goal 14: Life Below Water Conserve and sustainably use the oceans, seas and marine resources for sustainable development	YES	
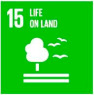	Goal 15: Life on Land Protect, restore and promote sustainable use of terrestrial ecosystems, sustainably manage forests, combat desertification, and halt and reverse land degradation and halt biodiversity loss	YES	
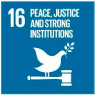	Goal 16: Peace, Justice and Strong Institutions Promote peaceful and inclusive societies for sustainable development, provide access to justice for all and build effective, accountable and inclusive institutions at all levels		YES
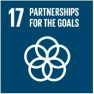	Goal 17: Partnerships for the Goals Strengthen the means of implementation and revitalize the Global Partnership for Sustainable Development		

* Common sustainable development goals for both standards.

**Table 2:** The managerial reasons for the modeling and application of the EMS and OHSMS cluster.

Managerial Information and Reasons:	EMS	OHSMS
existence of international standard	ISO 14001	ISO 45001
standard edition	3rd	1st
publication date	September 2015	March 2018
technical committee ISO/TC *	207/SC ** 1 Environmental management systems	283 Occupational health and safety management
ICS *** I.	13.020.10 Environmental management	13.100 Occupational safety. Industrial hygiene
ICS II.	03.100.70 Management systems	03.100.70 Management systems
number of pages	35	41
number of sustainable development goals	12	7
publication date	September 2015	March 2018
management systems standard	yes	yes
type of standard	A	A
including requirements	yes	yes
including guidance	yes	yes
structure of standard	harmonized (HS)	harmonized (HS)
management systems		
internal cycle	PDCA	PDCA
approach	process	process
reasoning	risk	risk
certifiable	yes	yes
applicable	any organization regardless of its size, type and activities	any organization irrespective of its size, type and activities
used	in whole or in part	in whole or in part
achievement of objectives	yes	yes
continual improvement of performance	yes	yes

* (TC) Technical Committee, ** (SC) Subcommittee, *** (ICS) International Classification for Standards.

**Table 3:** Number of requirements, {R}, in composed brackets, and documented information, DI, for the standards ISO 14001:2015 EMS and ISO 45001:2018 OHSMS, and their common {R} and DI.

**PDCA Cycle**	**Clause/Subclause/Sub^2^clause/Sub^3^clause**	**Number of** **{R** **}/DI**		**Common** **{R** **}/DI**
		**EMS**	**OHSMS**	
	4 Context of the organization			
P	4.1 Understanding the organization and its context	{2}	{2}	{2}
P	4.2 Understanding the needs and expectations of interested parties/* workers and other interested parties *	{3}	{3}	{3}
	4.3 Determining the scope of the EMS/* OHSMS *	{9}/1	{8}/1	{7}/1
	4.4 Environmental/* OHS * management system and its process(es)	{2}	{1}	{1}
	A partial summary of the 4th Clause:	{16}/1	{14}/1	{13}/1
	5 Leadership/* and worker participation *			
	5.1 Leadership and commitment	{9}	{13}	{8}
	5.2 (Environmental)/* OH&S * policy	{8}/1	{10}/1	{5}/1
	5.3 Organizational roles, responsibilities, and authorities	{3}	{5}/1	{3}
P	* 5.4 Consultation and participation of workers *		{20}	
	A partial summary of the 5th Clause:	{20}/1	{48}/2	{16}/1
P	6 Planning			
P	6.1 Actions to address risks and opportunities			
P	6.1.1 General	{12}/2	{13}/2	{12}/2
P	6.1.2 Environmental aspects/* 6.1.2 Hazard identification and assessment of risks and opportunities *	{8}/3		
P	* 6.1.2.1 Hazard identification *		{16}	
P	* 6.1.2.2 Assessment of OH&S risks and other risks to the OH&S management system *		{5}/2	
P	* 6.1.2.3 Assessment of OH&S opportunities and other opportunities for the OH&S management system *		{4}	
P	6.1.3 Compliance obligations/* 6.1.3 Determination of legal requirements and other requirements *	{4}/1	{5}/2	
P	6.1.4 Planning action	{6}	{8}	{4}
P	6.2 Environmental/* OH&S * objectives and planning to achieve them			
P	6.2.1 Environmental/* OH&S * objectives	{8}/1	{9}	{7}
P	6.2.2 Planning actions to achieve environmental/* OH&S * objectives	{6}	{8}/2	{6}
	A partial summary of the 6th Clause:	{42}/7	{68}/8	{29}/2
D	7 Support			
D	7.1 Resources	{1}	{1}	{1}
D	7.2 Competence	{5}/2	{4}/1	{4}/1
D	7.3 Awareness	{4}	{6}	{3}
D	7.4 Communication			
D	7.4.1 General	{8}/1	{12}/1	{8}/1
D	7.4.2 Internal communication	{2}	{2}	{2}
D	7.4.3 External communication	{1}	{2}	{1}
D	7.5 Documented information			
D	7.5.1 General	{2}/2	{2}/2	{2}/2
D	7.5.2 Creating and updating	{3}	{3}	{3}
D	7.5.3 Control of documented information	{2 ÷ 8} **	{2 ÷ 8} **	{2 ÷ 8} **
	A partial summary of the 7th Clause:	{28 ÷ 34} **/5	{34 ÷ 40} **/4	{26 ÷ 32} **/4
D	8 Operation			
D	8.1 Operational planning and control	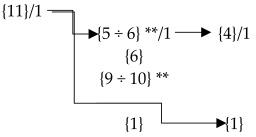		
D	* 8.1.1 General *			
D	* 8.1.2 Eliminating hazards and reducing OH&S risks *			
D	* 8.1.3 Management of change *			
D	* 8.1.4 Procurement *			
D	* 8.1.4.1 General *			
D	* 8.1.4.2 Contractors *		{6}	
D	* 8.1.4.3 Outsourcing *		{3}	
D	8.2 Emergency preparedness and response	{8}/1	{10}/2	{5}/1
	A partial summary of the 8th Clause:	{19}/2	{40 ÷ 42} **/3	{10}/2
C	9 Performance evaluation			
C	9.1 Monitoring, measurement, analysis and * performance * evaluation			
C	9.1.1 General	{10}/1	{13 ÷ 14} **/2	{9}/1
C	9.1.2 Evaluation of compliance	{5}/1	{6}/1	{5}/1
C	9.2 Internal audit			
C	9.2.1 General	{4}	{4}	{4}
C	9.2.2 Internal audit programme	{6}/1	{6}/1	{6}/1
C	9.3 Management review	{22}/1	{15 ÷ 16}/2	{12}/1
	A partial summary of the 9th Clause:	{47}/4	{46 ÷ 48} **/6	{36}/4
A	10 Improvement			
A	10.1 General	{2}	{2}	{2}
A	10.2 * Incident,* nonconformity and corrective action	{13}/3	{14 ÷ 17} **/2	{9}/2
A	10.3 Continual improvement	{1}	{5}/1	{1}
	A partial summary of the 10th Clause:	{16}/3	{21 ÷ 24} **/3	{12}/2
	Total summary, Figure 7:	{188 ÷ 194}/23	{271 ÷ 284}/27	{142 ÷ 148}/16

* Related to the standard ISO 45001:2018, OHSMS; ** interval of requirements depends on activities as applicable or as per the interested parties’ requirements.

## Data Availability

A part of the data supporting reported results in this research can be found on the web pages of the International Standard Organization. These links are publicly archived: https://www.iso.org/obp/ui/#iso:std:iso:14001:ed-3:v1:en (accessed on 17 January 2022); https://www.iso.org/obp/ui/#iso:std:iso:45001:ed-1:v1:en (accessed on 17 January 2022).
